# Pre-Diagnosis Dietary Pattern Differences in Australian Children with Inflammatory Bowel Disease: Exposure Across Ethnicities

**DOI:** 10.3390/nu18091313

**Published:** 2026-04-22

**Authors:** Nisha Thacker, Shoma Dutt, Emily C. Hoedt, Edward V. O’Loughlin, Clare E. Collins, Kerith Duncanson

**Affiliations:** 1 School of Health Sciences, College of Health Medicine and Wellbeing, The University of Newcastle, Newcastle, NSW 2308, Australia; 2 Food and Nutrition Research Program, Hunter Medical Research Institute, Newcastle, NSW 2305, Australia; 3 Department of Gastroenterology, The Children’s Hospital at Westmead, Sydney Children’s Hospital Network, Westmead, NSW 2145, Australia; 4 Children’s Hospital at Westmead Clinical School, Sydney Medical Program, University of Sydney, Sydney, NSW 2145, Australia; 5 NHMRC Centre of Research Excellence in Transforming Gut Health, The University of Newcastle, Newcastle, NSW 2308, Australia; 6 School of Biomedical Sciences and Pharmacy, College of Health Medicine and Wellbeing, The University of Newcastle, Newcastle, NSW 2308, Australia; 7 School of Medicine and Public Health, College of Health, Medicine and Wellbeing, The University of Newcastle, Newcastle, NSW 2308, Australia

**Keywords:** paediatric, ethnicity, plant food diversity, traditional diet

## Abstract

Background/Objectives: The pre-diagnosis dietary intake in newly diagnosed multi-ethnic paediatric inflammatory bowel disease (PIBD) is not well understood. This study aimed to describe the pre-diagnosis diet and environmental factors in children with newly diagnosed PIBD attending a single Australian tertiary children’s hospital. Methods: A pilot cross-sectional study was conducted from February 2022 to February 2023 involving children with newly diagnosed PIBD. Results: Of 56 children confirmed with PIBD, 54% had Crohn’s disease (CD)—mean ± SD age, 11.55 years ± 2.84—and 46% had Ulcerative Colitis (UC)—11.50 years ± 2.94 (45%, non-Caucasian). More Caucasians had an IBD family history (48.3% vs. 20%; *p* = 0.02 *). Non-Caucasian children demonstrated significantly lower mean serum vitamin D levels than Caucasian children (42.5 vs. 69 nmol/L; *p* ≤ 0.001 ***). Most children across ethnicities for both IBD subtypes had ‘regular’ intakes of red meat, whereas more Caucasian children had ‘regular’ intakes of processed/deli meat (72% vs. 39%; *p* = 0.02 *). A total of 64% of non-Caucasian children with CD reported a usual pre-diagnosis diet that differed from the traditional diet, compared to 42% with UC (*p* = 0.29). When eating out, fast foods were chosen regularly by most children with PIBD. Pre-diagnosis dietary intake data indicated that most with PIBD ‘rarely/never’ had whole-food sources of plant protein and had ‘infrequent’ intake of rice. Plant food diversity was low (mean 11 types/week). Conclusions: The significantly lower likelihood of IBD family history, along with relatively lower vitamin D levels, and the predominance of a Western-style dietary pattern among non-Caucasian children are compatible with the hypothesis that non-genetic factors may be important in PIBD, warranting further investigation into diet and environmental factors in this group. Further investigation of the pre-disease modifiable non-genetic factors contributing to the development of PIBD in the migrant population group is recommended. The finding across ethnicities of low pre-diagnosis plant food diversity was novel; however, due to the lack of healthy controls and the use of a novel but non-validated exposome tool, causality associations should be interpreted cautiously.

## 1. Introduction

The global incidence of paediatric inflammatory bowel disease (PIBD) is escalating [1,2]. The evolving epidemiology of PIBD demonstrates geographical variation [1,2]. Interestingly both the incidence and prevalence of PIBD have been increasing in countries with already reported high incidences, as well as emerging in countries where it was not previously reported. Furthermore, the age of diagnosis in PIBD has been reported to be decreasing [1]. Khan et al. recognised the impact of ethnicity/migration in PIBD [1] and reported that certain ethnicities (Jews, Arabs, and South Asian Canadians) seem to have a higher prevalence of IBD. One of the fascinating findings of a Canadian study showed a 14% drop in IBD risk with every decade increase in age at migration to a developed country [3], highlighting the possible significance of the time/age of exposure to the environmental and dietary factors (e.g., paediatric vs adult).

The environmental and dietary exposomes associated with PIBD possibly influence the gut microbiome and PIBD risk. Our recent meta-analysis on this topic highlighted associations between PIBD risk and exposure to ≥four antibiotic courses, passive smoking, not being breastfed, sugary drink intake, being a non-Caucasian child living in a high-income country and infection history [4]. When IBD subtype was explored, we found paediatric Crohn’s was associated with additional factors like socio-economic status, maternal smoking and history of atopic conditions, whereas paediatric UC was associated with additional factors like number of siblings and pet exposure. Dietary patterns in early years of life including breastfeeding and early childhood nutrition have a crucial role in shaping the gut microbiome, highlighting the need to investigate the influence of these factors on risk of PIBD.

The westernisation of lifestyle including the loss of traditional diets has been suggested as a likely contributing factor to increasing IBD incidence [1]. A diet pattern which is high in animal protein and saturated fat and low in fruit, vegetables and dietary fibre has been reported to increase IBD risk [5,6,7]. Ultra-processed foods (UPFs) and the additives in them (emulsifiers, thickeners, preservatives, flavour enhancers, whitening agents, artificial sweeteners) have been emerging in the literature as influencers of the IBD risk [8,9]. The proposed risk mechanism is through the promotion of proinflammatory intestinal microbiota, resulting in the breakdown of the mucosal layer and consequent increased intestinal permeability-associated activation of inflammatory pathways [10,11,12].

Dietary patterns determine the gut microbiome composition [13], which is recognisably altered in IBD [14]. Baseline pre-diagnosis diet patterns have been explored recently to evaluate the impact on baseline microbial diversity [15]. Emerging evidence suggests including whole plant foods, such as minimally processed vegetables, fruits, grains, nuts, seeds, lentils, legumes, herbs and spices, exerts immune regulatory and favourable gut microbiome-related effects [16]. The American Gut Consortium reported that eating more than thirty types of plant foods was associated with several putative short-chain fatty acid (SCFA) fermenters and a significant increase in alpha-diversity [17].

Australia has a fast-growing immigrant population including non-Caucasians, yet limited data is available on PIBD etiological factors. Children of immigrants are vulnerable to the host country’s food environment, where access to high-calorie nutrient-poor UPFs is higher. To our knowledge, no published studies have evaluated the pre-diagnosis dietary and environmental factors, including the impact of acculturation, in immigrant children with newly diagnosed IBD in a multi-ethnic Australian setting. Acculturation is usually described as a process of transition or cultural adaptation in terms of beliefs, attitudes, values and behaviours to a new/host country’s culture, often influencing lifestyle and dietary patterns.

The primary aim of this pilot cross-sectional study is to describe the pre-diagnosis early childhood characteristics, including the dietary patterns, among a multi-ethnic cohort of newly diagnosed paediatric IBD patients in a tertiary hospital in Australia. The focus was on the overall dietary pattern, UPF intake and plant food diversity, as well as early childhood factors like antibiotic exposure and breastfeeding history. The secondary aim is to explore potential differences by ethnicity and IBD subtype. The exploratory sub-group analysis studied includes ethnicity differences, IBD subtype differences, acculturation, and vitamin D levels.

## 2. Materials and Methods

### 2.1. Ethics Statement

This study was approved by the Human Research Ethics Committee of the SCHN (ETH number 2021/ETH11077). All families received information about the study and provided informed consent before enrolment. Children could provide assent. No benefits were given to the participants.

### 2.2. Study Cohort

Participants included children newly diagnosed with IBD whose parents consented to take part at the time of evaluation for IBD diagnosis. Sixty-seven children were evaluated for suspected PIBD.

### 2.3. Study Design

A cross-sectional survey was conducted prospectively from February 2022 to February 2023 at the Children’s Hospital Westmead (CHW), a tertiary children’s hospital in the Sydney Children’s Hospital Network (SCHN) in New South Wales, Australia.

Information collected during diagnostic work-up for IBD included self-reported ethnicity, diet, environment, and early childhood medical history. Data extracted from electronic medical records (eMRs) included a clinical, growth, and diagnostic work-up including endoscopy and histology findings. The specific blood tests included inflammatory markers like erythrocyte sedimentation rate (ESR), C-reactive protein (CRP) and nutritional markers. The stool test included an infectious screen as well as faecal calprotectin. The blood and stool samples were analysed at CHW’s accredited pathology laboratory. Diagnosis of IBD was based on imaging and endoscopic and histological assessment of intestinal mucosa. Children with newly diagnosed IBD were grouped into three subtypes: Crohn’s disease (CD), Ulcerative Colitis (UC) and IBD-Undifferentiated (IBD-U) as per established endoscopic and histological criteria [18]. Children with IBD-U were excluded to ensure diagnostic homogeneity. Disease severity was assessed as per ECCO-ESPGHAN guidelines [19,20] by disease activity scores as described in further detail in Supplementary Table S1.

### 2.4. Growth Measurements

Weight, height, and body mass index (BMI) data was extracted including percentile and BMI z-scores plotted on Centre for Disease Control (CDC) charts [21]. Participants were classified as overweight (>85–95% on BMI percentile) or obese (>95% on BMI percentile).

Malnutrition (undernutrition) was based on ICD-10 criteria (i.e., moderately or severely malnourished defined as a BMI z-score ≥ −2). Height and weight were measured at the hospital.

### 2.5. Dietary and Environmental Factors

In the absence of an existing comprehensive PIBD-specific validated dietary and environmental assessment tool to assess pre-disease dietary intake and environmental exposure, a customised survey was developed that included core food groups (based on Australian Dietary Guidelines) [22], UPF (for Australian paediatric population) [23], specific ingredients of concern/interest in IBD [8,24], diet diversity and other relevant data (Canadian Children IBD Network CIDsCaNN tools [25]) and acculturation factors. Parents were carefully and deliberately asked to recall and report dietary intake patterns prior to IBD diagnosis and were prompted to report intake before any symptom-based dietary changes were made prior to IBD diagnosis.

Diet diversity is defined as the number of different foods consumed over a given reference period [26]. Plant food intake diversity was assessed as per the definition by The American Gut Consortium as the number of unique plant species one consumes per week. Dietary herb and spice intake was collected but not included in counts due to very small portion sizes [17]. The frequency categories for food group intake used were: at least 1–2 days/week; at least 3 days/week; infrequent (≤1–2 days /week); rarely/never. This questionnaire was custom-designed for assessing different aspects of dietary patterns and has not been externally validated. The pre-disease questionnaires (Supplementary Table S2) had 42 questions.

This pilot study is descriptive and hypothesis-generating in nature.

### 2.6. Statistical Analysis

Statistical analyses were carried out using Stata software 18 (StataNow/BE 18.5 Mac-Apple Silicon, StataCorp LLC., 4905 Lakeway Drive, College Station, TX 77845-4512, USA). Descriptive statistics were used for demographic and clinical characteristics. Exploratory analyses evaluated potential associations between plant food variety by IBD subtype (CD vs. UC) and ethnicity (*t*-test), and between ethnicity and IBD subtype (chi-squared test). The small sample size and hypothesis-generating nature of the analysis means the results should not be viewed as definitive between-group differences but rather as descriptive. Continuous variables were analysed using independent-samples *t*-tests, which are generally considered to be robust to moderate departures from normality. Given the exploratory aims of the study, formal normality testing was not performed, as such tests are known to be unreliable and underpowered with small sample sizes. Non-parametric alternatives were not pursued as rank-based differences in medians were considered less interpretable than mean differences for the continuous variables reported here. Analysis results of *p* < 0.05 were considered statistically significant.

## 3. Results

### 3.1. Patient Demographics

**Figure 1:**
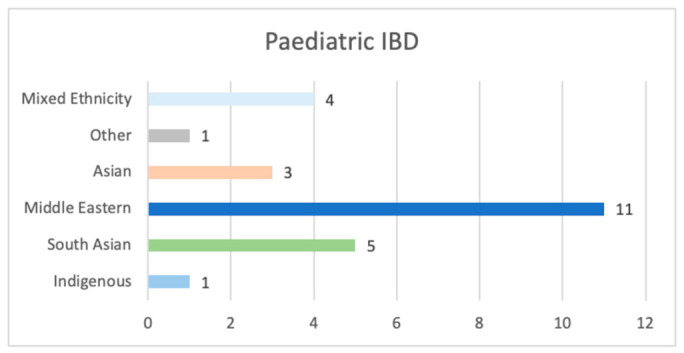
Paediatric IBD: non-Caucasian ethnicity sub-groups (*n* = 25).

A total of 56 children were included in the analysis. Of these, 54% had CD and 46% UC, 55% (*n* = 31) were Caucasian and 45% (*n* = 25) non-Caucasian, of which major sub-sets were Middle Eastern (20%), followed by South Asian (9%), as shown in Figure 1. The non-Caucasian ethnicity spread for UC (*n* = 14) and CD (*n* = 11) followed a similar trend of ethnicity spread as PIBD.

Table 1 presents demographics, nutritional markers and growth characteristics. Around two thirds of the children reported being born in Australia. Of those children born overseas (*n* = 7), four were Middle Eastern and two were from South Asia (India). Vitamin D levels were significantly lower in non-Caucasian children (42.5 vs. 69 nmol/L; *p* = <0.001 ***). More Caucasian (48% vs. 20%) children had an IBD family history (*p*= 0.02 *). The mean age of both CD and UC at diagnosis was 11 years with a male predominance. A total of 23% of children with CD and 16% with UC had a BMI above the healthy range. A total of 4% (1/25) in UC and 19% (5/26) in CD were underweight [27]. Endoscopic disease severity (Supplementary Table S6) for CD (SES, *p* = 0.48; NS) and UC (Mayo score, *p* = 0.14) were not associated with ethnicity. The majority of children with the CD phenotype (Supplementary Table S6) were between the ages of 10 and 17 years, with ileocolonic disease with non-stricturing and non-penetrating disease behaviour and no evidence of growth delay. Most of the children with the UC phenotype had pancolitis (Supplementary Table S6).

### 3.2. Pre-Diagnosis Early Childhood, Environmental and Socio-Economic Factors

Table 2 presents early childhood, environmental and socio-economic characteristics. Most children (89.3%) with IBD were initially breastfed; however, only around half were breastfed up to or beyond 12 months of age. Over half the children with IBD were born vaginally, with 28% more with CD born by caesarean delivery than those with UC (*p* = 0.02 *). A total of 41% had antibiotic exposure before two years of age, with no significant differences by IBD subtype or ethnicity. Around a quarter of fathers and mothers of children with IBD were an ex- or current smoker, and a non-significantly greater proportion of non-Caucasians with PIBD had a father who is an ex smoker or is a current smoker (*p* = 0.05). More Caucasian children had parents who reported the highest and second highest category of annual household income.

### 3.3. Pre-Diagnosis Exposure to Dietary Factors

**Figure 2:**
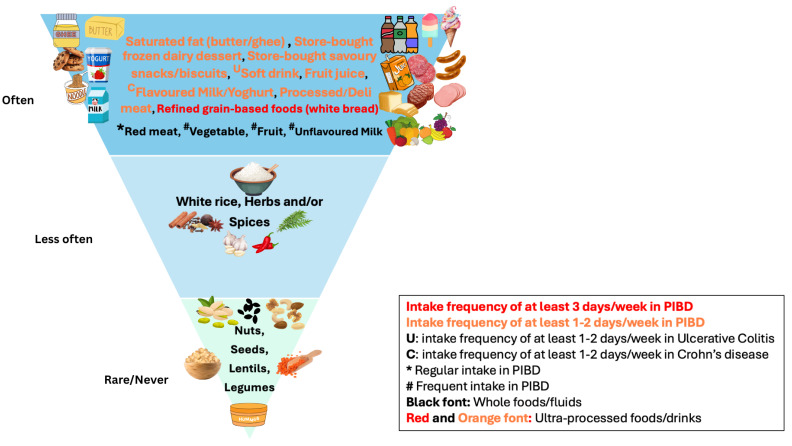
Pyramid representation of the frequency of the reported pre-diagnosis intake of various whole-foods and discretionary foods (ultra-processed foods/drinks) in the majority of the children with newly diagnosed PIBD.

The key findings about pre-diagnosis intake of food groups are summarised in Figure 2 from detailed data in Supplementary Table S3. The key dietary pattern findings for core food group intake are infrequent intake of rice, despite rice being the staple grain for the non-Caucasian sub-group, and rare intake of plant protein-rich foods like legumes/lentils and nuts/seeds in both the CD and UC groups. However, these data reflect the frequency of food/fluid intake, not portion sizes or comparator data, and therefore should be interpreted as descriptive data.

#### 3.3.1. Pre-Diagnosis Dietary Pattern

The majority (>70%) across ethnicity subtypes (as well as IBD subtypes) reported at least 1–2 days/week intake of red meat. More children with CD reported at least 1–2 days/week intake of flavoured yoghurt/milk (66% vs. 50%; *p* = 0.04 *), whereas intake was similar between ethnicity subtypes with more than half having flavoured dairy at least 1–2 days/week. Caucasian children were 28% more likely to report infrequent (<1–2 days/week) intake of staple grains like rice, compared to non-Caucasian children (71% vs. 43%, *p* = 0.009 *).

However, based on IBD subtypes, more than half the children reported infrequent intake of rice in both CD and UC. Most of the children with IBD consumed vegetables (76%) and fruits (82%) at least 3 days/week. As frequencies but not quantities were reported, it was not possible to report on the adequacy of fibre or fruit and vegetable intake.

Around three quarters of Caucasians ‘rarely/never’ had lentil/legumes (a plant protein-rich food group) and more than half of non-Caucasian children ‘rarely/never’ ate nuts/seeds (another plant protein-rich food group). More than half the children in both the CD and UC groups ‘rarely/never’ had pre-diagnosis plant protein-rich foods (Supplementary Table S3).

#### 3.3.2. Discretionary Foods and Eating Away from Home

A non-significantly higher proportion of non-Caucasian than Caucasian children drank soft drinks (57% vs. 43%; *p* = 0.44) and juice (65% vs. 50%; *p* = 0.44) at least 1–2 days/week (Supplementary Table S3). Between ethnicities, 33% more Caucasian children ate processed/deli meat at a frequency of at least 1–2 days/week (*p* = 0.02 *).

Eating out was common, with around half reporting a pre-diagnosis eating-out frequency of 1–2 days/week (*n* = 23/48) followed by 35% for 1–3 days/month (*n* = 17/48). When eating out, pre-diagnosis intake of fast foods at least 50% of time was reported by more than half of the children across both IBD subtypes. Pre-diagnosis fast-food intake for at least 50% of the time when eating outside of home was present slightly more in non-Caucasian children (61% vs. 56%; *p* = 0.40).

The majority in both CD and UC groups reported a pre-diagnosis intake frequency of at least 1–2 days/week of saturated fat and store-bought dairy desserts. More than half in both CD and UC groups reported a pre-diagnosis frequency of at least 1–2 days/week of intake of processed/deli meat and around a third reported 3 days per week or more. Almost all reported having store-bought savoury snacks/biscuits at least 1–2 days/week. More than half of the children with UC reported at least 1–2 days/week intake of soft drinks and fruit juice.

#### 3.3.3. Pre-Diagnosis Intake of Traditional vs. Westernised Meals in Non-Caucasian Children

Similarly to the findings in non-Caucasian parents, just over half (*n* = 12/23) of non-Caucasian parents reported that their child’s usual pre-disease diet differed from their traditional cultural diet. Deviation from traditional dietary patterns was reported in 64% of children with CD in contrast to 42% of those with UC (*p* = 0.29), which did not reach statistical significance.

Furthermore, when exploring specific meal occasions, breakfast, snacks and desserts were the most frequently reported as meals in which traditional foods were never/sometimes consumed (Supplementary Table S7). Of the 23 non-Caucasian children, more than 80% ‘*never*/*sometimes*’ had a traditional breakfast, more than 90% ‘never/sometimes’ had traditional snacks and more than 90% ‘never/sometimes’ had traditional desserts. The majority of the non-Caucasian children with UC ‘often/very often’ had traditional foods at dinner, compared to only one quarter with CD. Similarly, more non-Caucasians with UC (50%) ‘often/very often’ had a traditional lunch, compared to only 9% with CD.

#### 3.3.4. Pre-Diagnosis Plant Food Diversity

The mean number of plant foods eaten per week was 11.5 ± 5.41, with no statistically significant difference in plant food diversity by IBD subtypes (*p* = 0.33) or ethnicities (*p* = 0.68) (Supplementary Table S4). Middle Eastern children had the lowest pre-disease intake of the number of different plant foods each week (9.3 ± 5.2), whereas South Asians reported the highest (16.4 ± 7.6) (Supplementary Table S5).

### 3.4. Acculturation and Parental Factors Amongst Non-Caucasians

A total of 43% of mothers (*n* = 22/51) and 39% of fathers (*n* = 20/51) self-identified as non-Caucasian. Among non-Caucasian parents, just over half reported that their own usual diet, prior to their child’s recent IBD diagnosis, differed from their traditional cultural diet. Of these, 70% of parents of children diagnosed with CD and 40% of parents of children with UC reported their own diet deviated from traditional meals (Supplementary Table S7).

Out of 22 non-Caucasian children, the majority reported their child often/very often spoke with family members in English and almost all reported using English when communicating with friends.

## 4. Discussion

This study compared self-reported pre-disease exposomes and clinical data of children with newly diagnosed IBD in an ethnically diverse tertiary hospital setting in NSW, Australia. Differences by ethnicity and IBD subtype (CD vs. UC) were evaluated. There was an even distribution of Caucasian and non-Caucasian children. Notable environmental characteristics of the study sample included a substantial proportion from a high family-income category, with close to half exposed to antibiotics before the age of two years. While the majority had been breastfed, only around half continued to 12 months of age or longer.

Non-Caucasian children had significantly lower blood vitamin D levels prior to diagnosis, whereas having IBD family history was significantly more common among Caucasians. The findings related to vitamin D should be considered within the context of potentially confounding factors like skin pigmentation, clothing practices, seasonal variation and sun exposure and cannot be attributed solely to dietary intake or sun exposure. Overall, dietary intake data indicated Western-style patterns, low diversity in plant foods and regular intake of UPFs. Furthermore, even though we found the self-reported intake of vegetables and fruit on a weekly basis, the quantitative intake was not assessed due to the nature of the tool used, limiting the ability to determine the intake of actual fibre/other plant compounds like polyphenols from these food groups.

This study provides exploratory insights and highlights exposomes that need further investigation as the methodological limitations, including small sample size, precludes the analysis of causal relationships.

Few characteristics differed by IBD and ethnicity subtype. Significantly more non-Caucasian fathers were ex- or current smokers. Having a higher proportion of Caucasian children in the CD group and non-Caucasians in the UC group aligns with several studies [28,29,30,31,32]. It has been hypothesised that second-generation immigrants are a sub-population at higher risk of IBD in industrialised countries [33]. Having a family history of IBD was more common in Caucasians, aligning with some paediatric studies [32,34,35,36] but differing from another [33]. The lower likelihood of a family history of IBD in non-Caucasians in parallel with rising IBD incidence in non-Caucasian immigrants raises the possible influence of environmental and lifestyle factors on disease pathogenesis [29,37,38]. Migration to developed countries has previously been proposed to be associated with triggering IBD risk [1] by exposing children in early life [3] to environmental and dietary exposomes (e.g., access to and affordability of UPFs).

Overall, the mean BMI z-score was in the healthy weight category. Blood vitamin D levels were within the reference range, although non-Caucasians had significantly lower blood levels of vitamin D (42.5 vs. 69 nmol/L). The findings related to blood levels of vitamin D for Caucasian children are similar to those reported in the Australian Health Survey (69 nmol/L) [39]. It is well-recognised that having dark skin with minimal sun exposure increases the risk of vitamin D deficiency. Whether lower vitamin D levels are a cause or consequence of PIBD in non-Caucasian children remains unclear. This immunoregulatory role of vitamin D [40] warrants further exploration in future mechanistic studies.

More than half of those with PIBD were born by vaginal delivery; however, children with CD had significantly higher caesarean delivery rates, which aligns with the findings of a meta-analysis [41]. Similarly, early life exposure to antibiotics is known to have a lifelong impact on the microbiome [42] due it coinciding with an infant’s gut immune and microbiome maturation phase. Findings that about 40% had been exposed to antibiotics before the age of two years align with an Italian longitudinal study [43] and a UK study on PIBD [44], highlighting the amplified influence of early childhood years on the gut microbiome [45,46].

Most children across IBD subtypes were breastfed; however, of those who were, around half were breastfed for 12 months or more. Reviews on IBD [46,47] and PIBD [4,47,48] suggest there may be a stronger protective role of being breastfed for at least 12 months [47,49]. The gut microbiome of formula-fed infants reaches its maximum maturity and diversity at three months of age, whereas in breastfed infants it continues to mature up to 12 months of age. This suggests that breastfeeding exclusivity and duration promote a more gradual and sustained gut microbiome diversity and maturity compared to the earlier maturation observed in non-breastfed infants [50].

Although most of the parents in the current study reported never smoking, more non-Caucasian fathers were an ex- or current smoker. Passive smoking was significantly associated with increased PIBD risk in our recent meta-analysis [4]. It is possible that a relatively short duration of passive smoking exposure in children is sufficient to influence the microbiome composition and/or disrupt immune responses [51].

Socio-economic status (SES) based on annual household income indicated a predominance of high-income categories across IBD subtypes and ethnicities. The low proportion of low-income families suggests that the included families were representative of the developed country income distribution. Higher SES has been reported to be significantly associated with paediatric CD risk [4]. Having more disposable income is likely to influence lifestyle including dietary patterns given the affordability of and accessibility to UPFs, which are likely to have an unfavourable impact on the gut microbiome, a well-recognised factor in IBD pathogenesis [14,52]. This is consistent with our finding that more than half of the children with PIBD ‘often’ had fast foods pre-diagnosis.

Pre-diagnosis dietary patterns in this study were inconsistent with national recommendations (Australian Dietary Guidelines [22]) across reported food groups. The ‘*infrequent*’ intake of fibre-rich whole plant foods (includes plant protein-rich foods like legumes/lentils, nuts, and seeds and staple grains like rice), at least 1–2 days/week intake of discretionary foods, and at least 3 days/week intake of refined grains collectively may play a role in increasing the risk of host intestinal mucus layer depletion by microbes, thereby increasing immune activation due to compromised gut barrier integrity [53]. Our novel finding of pre-diagnosis sub-optimal plant food diversity (i.e., intake of eleven different whole plant foods per week) across ethnicities was interesting, as beneficial effects on the gut microbiome have been reported when intake reaches thirty different plant foods per week or more [17]. Deliberate efforts were made during the dietary intake interview to collect dietary data prior not just to IBD diagnosis but also to making any changes in diet in response to undiagnosed IBD symptoms. However, the influence of early or prodromal symptoms cannot be eliminated; therefore, reverse causality cannot be dismissed.

The dietary intake pattern of many children indicated dietary acculturation. More than half of non-Caucasian parents reported that their own usual diet differed from their traditional diets, indicating a change, over generations, in exposure to traditional diets, consistent with the acculturation process. The dominant non-Caucasian ethnicities in this study were Middle Eastern and South Asian, where the traditional diet typically includes regular consumption of whole plant foods like wholegrains, rice, legumes/lentils, and nuts/seeds and has minimal intake of processed meat [54,55]. Notably, most non-Caucasian children in this study rarely consumed staple plant protein-rich foods and regularly consumed animal protein foods like red and processed meats. This is a shift from traditional diet patterns with likely subsequent alterations in intake of associated nutrients, including total fibre and different fibre types including resistant starch, unsaturated fats, and polyphenols. Together, these findings further demonstrate the need for well-powered studies to investigate pre-diagnostic dietary patterns in PIBD, with a focus on traditional diets.

Our findings align with a recent large multinational, prospective cohort study involving adults including immigrants, conducted in 21 low-, middle- and high-income countries that included Middle Eastern and South Asian regions [56]. The study reported the mean servings/week of individuals with IBD as 2.2 for processed meat, 1.5 for soft drinks, and 4 for UPFs, which was far greater than the intake reported in the country of origin.

The current findings may have implications relating to the association of acculturation and the development of PIBD. The results related to acculturation measures should be interpreted as preliminary because the measures used are simplified. Therefore, while the observations of deviation from cultural norms as a driver of IBD pathogenesis are plausible, it is not possible to develop robust interpretations and conclusions. Of note, in the absence of a healthy comparator group, it was not possible to assess if these dietary patterns and acculturation-related shifts were replicated or differed from those in children in the community without IBD.

Collectively, the findings of lower vitamin D levels among non-Caucasian children, low intake and diversity of whole plant foods, regular intake of UPFs and drinks and evidence of dietary acculturation may represent converging exposures relevant to the gut microbial ecosystem as well as gut barrier integrity. Vitamin D plays a well-recognised role in immune modulation/homeostasis [57] and lower levels may influence mucosal immune responses. Simultaneously, infrequent intake of various fibre-rich whole plant foods and low plant food diversity may reduce the overall fibre intake, the different types of fibre consumed and the various polyphenols that are found in plant foods—all of which have been recognised to have prebiotic effects. This may potentially limit short-chain fatty acid production, which is important for colonocyte nourishment as well as for protection of the mucus layer. Furthermore, limited plant food diversity may lead to less microbial diversity [17].

The observed shift from traditional dietary patterns—particularly among non-Caucasian families—toward regular intake of red/processed meat and UPFs may further influence the metabolites produced by the microbes.

The shift from traditional diets (which are typically rich in whole plant foods including wholegrains, legumes and nuts/seeds and regional and seasonal fruits and vegetables) towards Western/non-traditional diet patterns is characterised by nutrient-poor but energy- and additive/preservative-dense foods and drinks. These often tend to be high in refined carbohydrates, calories and saturated/trans-saturated fat intake and processed animal products, resulting in low fibre intake and plant food diversity, thereby creating a progressive shift towards an altered nutrient intake. Such dietary transitions have been shown to adversely affect the gut microbiome. To add further to the insult from an altered nutrient intake, additives like preservatives, emulsifiers, colours and artificial sweeteners often present in UPFs have been associated with detrimental effects on the gut microbiome [58]. Lastly, excess calorie intake, which is a common characteristic of westernised diet patterns, may contribute to excess adiposity, which is increasingly being recognised to be linked with systemic low-grade inflammation [59,60]. Together these highlight biologically plausible pathways through which acculturation and its associated impact on dietary patterns may influence gut microbiome composition and the metabolites produced, although this pathway remains complex and not fully understood.

Whilst dietary causality cannot be inferred from this study design, these cumulative dietary and environmental patterns provide biologically plausible hypotheses for future prospective studies investigating the role of dietary acculturation, micronutrient status and plant food diversity in paediatric IBD pathogenesis.

The strengths of this study include the presentation of pre-diagnosis dietary and environmental exposomes in a well-defined ethnically diverse group of children with newly diagnosed IBD, providing early childhood exposure insights in the Australian context. This contributes to the understanding of the potential role of the exposome and can help inform dietary guidance for reducing the risk of PIBD. This study was designed with an intention to move away from single-nutrient associations towards dietary patterns, plant food diversity and traditional-vs-western food transitions—a novel angle to explore the exposome context in PIBD. The use of hospital records to extract clinical, growth and laboratory markers ensured validated extraction methods.

This study also has limitations that need to be acknowledged. Firstly, the cross-sectional study design and lack of a healthy control group preclude any inference of causality between dietary pattern and IBD subtype/ethnicity. Secondly, the dietary and environmental factor-related data collection tool was not an externally validated tool and involved parent-reported recall, which may not accurately reflect habitual intake. Therefore, recall inaccuracy and recall bias cannot be eliminated. Additionally, although parents were prompted to report intake prior to changes made to the diet in response to IBD symptoms, the residual influence of early symptoms cannot be eliminated. The dietary assessment tool was based on frequency rather than quantity and consequently the data does not reflect the actual intake of nutrients including fibre. Therefore, even though frequency of fibre-containing foods was reported, total fibre and fibre types were not able to be measured. Different types of fibres are increasingly recognised in pre-clinical and emerging clinical studies to influence beneficial microbial metabolite production including short-chain fatty acids and thereby affect mucosal integrity [61]. The results reflect dietary patterns rather than nutritional quantification. Thus, these dietary findings need to be interpreted cautiously in terms of nutrient exposure. As a result, mechanistic implications cannot be inferred from this dataset. Lastly, the relatively small sample size limits the statistical power of the study and may limit the ability to detect modest associations between dietary exposures and clinical/ethnic variables. The small sample size also may increase the risk of type 1 error.

The findings presented here should be interpreted as exploratory as no adjustment for multiple testing was made. Furthermore, the sub-group analyses stratified by ethnicity and disease subtype were underpowered and should therefore be interpreted cautiously. These findings should be interpreted as descriptive observations which will generate hypotheses for future prospective studies incorporating healthy controls.

## 5. Conclusions

In conclusion, this study is the first of its kind describing self-reported pre-diagnosis dietary and environmental exposomes in PIBD in the Australian setting. There were some notable differences between Caucasians and non-Caucasians and IBD subtypes. The lower likelihood of a positive IBD family history along with relatively lower vitamin D levels and predominance of a Western-style eating pattern among non-Caucasian children are compatible with the hypothesis that non-genetic factors may be important in PIBD and warrant further investigation. The novel findings of low pre-diagnosis plant food diversity and rare intake of plant protein-rich foods pre-diagnosis across ethnicities support the plausibility of cultural dietary transitions as a driver of PIBD but require substantiation in robustly designed prospective studies to establish causation. However, the absence of healthy comparator groups in this observational cohort limits the ability to determine if this trend reflects the diet of urban Australian children or is specific to PIBD. Therefore, causal inferences cannot be made, and these findings should be interpreted as hypothesis-generating in nature instead of as confirmatory findings.

A validated PIBD-specific tool that integrates assessment of the dietary and environmental exposomes along with the acculturation-driven dietary transitions in immigrants is needed to characterise and address PIBD risk factors. Further research on the cultural shift in immigrant families, especially among Middle Eastern and South Asian migrants in Australia, would help to understand the interplay of environment and acculturation in PIBD pathogenesis and inform potential preventative strategies.

## Figures and Tables

**Table 1:** Demographics, nutritional markers and growth characteristics.

	Caucasian	Non-Caucasian	*p* Value	Ulcerative Colitis	Crohn’s Disease	*p* Value
***n*** ** = 56**	*n* = 31 (55.4%)	*n* = 25 (44.6%)		*n* = 26 (46.4%)	*n* = 30 (53.6%)	
**Age at IBD diagnosis** (mean ± SD)	11.5 ± 2.95 *n* = 30	11.5 ± 2.81 *n* = 23	0.98	11.50 ± 2.94 *n* = 26	11.55 ± 2.84 *n*= 27	0.94
**Ethnicity**	n/a	n/a				
Non-Caucasian				14 (53.8%)	11 (36.7%)	0.19
Caucasian				12 (46.2%)	19 (63.3%)	
**Non-Caucasian ethnicity sub-groups**	n/a	n/a				
Indigenous				0	1 (3.3%)	
South Asian				3 (11.5%)	2 (6.7%)	
Middle Eastern				7 (26.9%)	4 (13.3%)	
Asian				1 (3.8%)	2 (6.7%)	
Other				1 (3.8%)	0	
Mixed Ethnicity				2 (7.7%)	2 (6.7%)	
**Gender**						
Male	20 (64.5%)	17 (68%)	0.78	16 (61.5%)	21 (70%)	0.50
**No IBD family history**				19 (73.1%)	17 (56.7%)	0.20
**Nutritional markers** (mean ± SD)						
Vitamin D (51–250 nmol/L) ^$^	69.0 ± 22.9 (*n* = 16)	42.5 ± 18.7 (*n* = 19)	**0.0009**	53.5 ± 28.3 (*n* = 20)	56.2 ± 18.9 (*n* = 15)	0.73
Vitamin B12 ( ≥ 59 pmol/L) ^$^	141.6 ± 133.5 (*n*= 15)	207.7 ± 237.3 (*n* = 16)	0.34	142.1 ± 110.3 (*n* = 17)	216.5 ± 261.8 (*n*= 14)	0.33
Iron (9–24 umol/L) ^$^	8.8 ± 6.7 (*n* = 20)	5.9 ± 3.3 (*n* = 17)	0.09	7.6 ± 6.6 (*n* = 20)	7.3 ± 4.2 (*n* = 17)	0.86
Zinc (10–18 umol/L) ^$^	16.4 ± 7.0 (*n* = 7)	12.8 ± 7.6 (*n* = 7)	0.38	13.6 ± 7.0 (*n* = 8)	16.0 ± 8.0 (*n* = 6)	0.57
**BMI percentile (CDC ^a^)** (mean ± SD)	34.3 ± 30.9	40.6 ±33.7	0.49	41 ± 32.8	33.2 ± 31.2	0.38
**BMI z-score** (mean ± SD)	−0.75 ± 1.4 (*n* = 30)	−0.51 ± 1.4 (*n* = 23)	0.55	−0.44 ± 1.36 (*n* = 26)	−0.84 ± 1.48 (*n*= 27)	0.30
**BMI percentile**			0.19			0.42
>85–95% BMI ^b^ percentile (**Overweight**)	4 (14.2%) (*n* = 28)	2 (8.6%) (*n* = 23)		3 (12%) (*n* = 25)	3 (11.5%) (*n* = 26)	
>95% BMI percentile (**Obesity**)	1(3.5%) (*n* = 28)	3(13%) (*n*= 23)		1 (4%) (*n* = 25)	3 (11.5%) (*n* = 26)	

^$^ Reference value; ^a^ CDC, Centers for Disease Control; ^b^ BMI, body mass index; nmol/L, nanomoles per litre; pmol/L, picomoles per litre; µmol/L, micromoles per litre. Note: For some variables, data were not available for all participants; therefore, sample sizes (*n*) are smaller than the total study population. **Bold font** for *p*-values indicates statistically significant *p*-values (*p* < 0.05).

**Table 2:** Pre-diagnosis early childhood, environmental and socio-economic factors.

	Caucasian	Non-Caucasian	*p* Value	Ulcerative Colitis	Crohn’s Disease	*p* Value
***n*** ** = 56**	31 (55.4%)	25 (44.6%)		26 (46.4%)	30 (53.6%)	
**Number of siblings** (median, ^f^ IQR)	1.5 (1–3)	2 (1–3)		2 (1–3)	2 (1–2)	
**Mode of birth/delivery**						
Caesarean	9 (29%)	8 (32%)	0.81	4 (15.3%)	13 (43.3%)	**0.02**
Vaginal	22 (70.9%)	17 (68%)		22 (85%)	17 (56.6%)	
**Antibiotic exposure at birth**	4 (13.3%)	1 (4.1%)	0.24	2 (7.6%)	3 (10.7%)	0.70
**Antibiotic exposure under 2 years of age**	12 (41.3%)	10 (41.6)	0.98	11 (45.8%)	11 (37.9%)	0.56
**Breastfed**	28 (90.3%)	22 (88%)	0.78	22 (84.6%)	28 (93.3%)	0.29
**Breastfed** ≥ **12 months of age**	11 (39%)	11 (50%)	0.75	10 (62.5%)	12 (57.1%)	0.74
**Frequency of fast-food type of dine-in/takeaway meals**						
50% of the time	4 (14.8%)	7 (30.4%)	0.40	4 (18.2%)	7 (25%)	0.46
<50% of the time	12 (44.4%)	9 (39.1%)		8 (36.4%)	13 (46.4%)	
>50% of the time	11 (40.7%)	7 (30.4%)		10 (45.5%)	8 (28.6%)	
**Mother’s smoking history**						
Ex-smoker	7 (25%)	2 (8.7%)	0.18	4 (18.2%)	5 (17.2%)	0.95
Never	20 (71.4%)	18 (78.2%)		16 (72.7%)	22 (75.9%)	
Yes	1 (3.5%)	3 (13%)		2 (9.1%)	2 (6.9%)	
**Father’s smoking history**						
Ex-smoker	4 (14.2%)	2 (8.7%)	0.05	4 (18.2%)	2 (6.9%)	0.47
Never	22 (78.5%)	14 (60.8%)		14 (63.6%)	22 (75.9%)	
Unsure	1 (3.5%)	0		0	1 (3.4%)	
Yes	1 (3.5%)	7 (30.4%)		4 (18.2%)	4 (13.8%)	
**Age (years) at first episode of food poisoning**	4.9 ± 2.4 (*n* = 10)	7.1 ± 4.5 (*n* = 6)	0.30	5.50 ± 3.00 (*n* = 4)	5.87 ± 3.69 (*n* = 12)	0.84
**Age (years) at first episode of ear infection**	1.5 ± 0.8 (*n* = 6)	3.0 ± 1.4 (*n* = 2)	0.37	1.33 ± 0.76 (*n* = 3)	2.30 ± 1.20 (*n* = 5)	
**Age (years) at tonsillectomy**	4.6 ± 0.5 (*n* = 3)	6.3 ± 4.0 (*n* = 3)	0.5	7.50 ± 3.53 (*n* = 2)	4.50 ± 2.08 (*n* = 4)	
**Total annual household income**			0.10			
$26,000–$33,799/year	1 (3.5%)	0		1 (4.5%)	0	0.53
$52,000–$64,999/year	0	3 (13%)		2 (9.1%)	1 (3.4%)	
$65,000–$77,999/year	0	1 (4.3%)		1 (4.5%)	0	
$91,000–$103,999/year	2 (7.1%)	2 (8.7%)		2 (9.1%)	2 (6.9%)	
$104,000–$155,999/year	9 (32.1%)	5 (21.7%)		5 (22.7%)	9 (31.0%)	
$156,000 or more/year	14 (50%)	9 (39.1%)		9 (40.9%)	14 (48.3%)	
Don’t know	0	3 (13%)		2 (9.1%)	1 (3.4%)	
Don’t want to answer	2 (7.1%)	0		0	2 (6.9%)	

^f^ IQR: Interquartile Range. Note: For some variables, data were not available for all participants; therefore, sample sizes (*n*) are smaller than the total study population. **Bold font** for *p*-values indicates statistically significant *p*-values (*p* < 0.05).

## Data Availability

The original contributions presented in this study are included in the article/Supplementary Materials. Further inquiries can be directed to the corresponding authors.

## Supplementary Materials

The following supporting information can be downloaded at: https://www.mdpi.com/article/10.3390/nu18091313/s1, Table S1: Disease severity and phenotype; Table S2: Pre-diagnosis diet—environment form; Table S3: Pre-diagnosis food frequency; Tables S4: Association between number of different plant foods consumed per week: (a) Paediatric IBD subtype (*t*-test) and (b) Ethnicity (*t*-test); Table S5: Number of different plant foods intake per week based on paediatric IBD subtype and ethnicity; Table S6: Clinical characteristics; Table S7: Dietary Acculturation factors.

## Abbreviations

The following abbreviations are used in this manuscript:

PIBD/pIBD	Paediatric inflammatory bowel disease
CD	Crohn’s disease
UC	Ulcerative colitis
SCFA	Short-chain fatty acid
UPFs	Ultra-processed foods
ESR	Erythrocyte sedimentation rate
CRP	C-reactive protein
eMR	Electronic medical record
IBD-U	IBD-undifferentiated
BMI	Body mass index
CDC	Centre for Disease Control
