# Supplementary material for: Pre-Diagnosis Dietary Pattern Differences in Australian Children with Inflammatory Bowel Disease: Exposure Across Ethnicities
Source: Nutrients. 2026 Apr 22;18(9):1313. doi: 10.3390/nu18091313 (PMC13165386; doi:10.3390/nu18091313)
Supplement: Supplementary file 1 [file nutrients-18-01313-s001.zip › Table S1-Disease severity and phenotype.docx]

**Supplementary Table S1. Disease severity and phenotype**

**CD severity:**

1. **Paediatric Crohn’s Disease Activity Index (PCDAI) score**

- abdominal pain, stool pattern, and general well-being (0, 5 or 10 each)
- presence of extraintestinal manifestations (0, 5, or 10)
- physical examination findings (abdomen, peri-rectal disease, 0, 5 or 10 each)
- weight and height (0, 5 or 10 each)
- ESR and haematocrit (0. 2.5 or 5 each), serum albumin (each 0, 5 or 10)
- Validated to accurately reflect disease activity physician-assessed global assessment.
- PCDAI score of 30-37.5 represents disease of moderate activity and 40-100 of severe activity.

1. **Endoscopic severity (SES):**

Four components scored for each segment of gastrointestinal tract: presence and size of ulcer, narrowing, surface involved by disease & ulceration. The total score ranges from 0 to 15 or more. A higher score indicates worse disease severity.

- **SES 0–2**: Remission (provided that no sub-score for each single parameter is >1)
- **SES 3–6**: mild endoscopic activity
- **SES 7–15**: moderate endoscopic activity
- **SES >15**: severe endoscopic activity

**UC severity:**

1. **Paediatric Ulcerative Colitis Activity Index (PUCAI) score:**

- abdominal pain, stool consistency, and activity level (0, 5 or 10 each), nocturnal bowel movements (0 or 10)
- rectal bleeding (0, 10, 20 or 30) and number of stools per 24 hours (0, 5, 10 or 15)
- PUCAI score of 35-60 represents disease of moderate activity and >=65 of severe activity.

1. **Endoscopic severity (Mayo score):**

- Scoring based on mucosal appearance including erythema, bleeding and ulceration. The total score ranges from 0 to 3. A higher score indicates worse disease severity.
- Mayo score 0: normal mucosa or inactive disease
- Mayo score 1: mildly activity (erythema, decreased vascular pattern, mild friability)
- Mayo score 2: moderate activity (marked erythema, lack of vascular pattern, friability, erosions)
- Mayo score 3: severe activity (spontaneous bleeding, large ulcerations)

**CD phenotype (Paris classification):**

- Age at Diagnosis: A1a representing child age of < 10 years and A1b representing 10-17 years old children
- Location involved

L1: Distal 1/3 ileum +limited caecal disease

L2: Colonic

L3: Ileocolonic

L4a: Upper disease proximal to Ligament of Treitz

L4b: Upper disease distal to Ligament of Treitz and proximal to distal 1/3 ileum

- Behaviour of disease

B1: Non stricturing & non penetrating

B2: Stricturing

B3: Penetrating

B2B3: Both, stricturing & penetrating disease

P: Perianal disease modifier

- Growth (whether affected)

G_0_ = No evidence of growth delay

G_1_ = Growth delay

UC phenotype (Paris classification):

- Extent of the disease:

E1: Ulcerative Proctitis

E2: Left Sided UC (distal to splenic flexure)

E3: Extensive (hepatic flexure distally)

E4: Pancolitis (Proximal to hepatic flexure)

S0: never severe* (* Severe defined by PUCAI > or = 65)

S1: ever severe* (* Severe defined by PUCAI > or = 65)

**Study title:** **Pre-Diagnosis Dietary Pattern Differences in Australian Children with Inflammatory Bowel Disease: Exposure Across Ethnicities**

Nisha Thacker^1,2^ **M. Nutr. & Diet**.[Nisha.Thacker@uon.edu.au](mailto:Nisha.Thacker@uon.edu.au) (corresponding author)

Shoma Dutt^3,4^ **PhD** [shoma.dutt@health.nsw.gov.au](mailto:shoma.dutt@health.nsw.gov.au)

Emily C. Hoedt^5,6^ **PhD** [Emily.Hoedt@newcastle.edu.au](mailto:Emily.Hoedt@newcastle.edu.au)

Edward V O’Loughlin^3^ **MD** [ted.oloughlin@health.nsw.gov.au](mailto:ted.oloughlin@health.nsw.gov.au)

Clare E Collins^1,2^ **PhD** [clare.collins@newcastle.edu.au](mailto:clare.collins@newcastle.edu.au)

Kerith Duncanson^2,5,7^ **PhD** [kerith.duncanson@newcastle.edu.au](mailto:kerith.duncanson@newcastle.edu.au) (corresponding author)

The Children’s Hospital Westmead, Sydney Children’s Hospital Network, Australia
