# Supplementary material for: Pre-Diagnosis Dietary Pattern Differences in Australian Children with Inflammatory Bowel Disease: Exposure Across Ethnicities
Source: Nutrients. 2026 Apr 22;18(9):1313. doi: 10.3390/nu18091313 (PMC13165386; doi:10.3390/nu18091313)
Supplement: Supplementary file 1 [file nutrients-18-01313-s001.zip › Table S2-Pre-Diagnosis Diet.pdf]

Date: **Identifier:**

INITIAL GASTROENTEROLOGY CONSULT - BASELINE DIETARY AND ENVIRONMENTAL FACTORS- FORM D

What is the child's mother's ethnic background?

Caucasian *non-Caucasian* *(if Caucasian skip "description of traditional diet "question below)*

What is the child's father's ethnic background?

Caucasian *Non-Caucasian* *(if Caucasian skip "description of traditional diet "question below)*

Environmental – Dietary Factors

How would you describe **child's mother's traditional diet** (i.e., what cuisine/culture type)? *(Applicable if selects non-Caucasian ethnic background)*

How would you describe **child's father's traditional diet** (i.e., what cuisine/culture type)? *(Applicable if selects non-Caucasian ethnic background)*

How does **child's mother's traditional diet** compare to a westernised or typical Australian diet? *Similar/Different*

How does **child's father's traditional diet** compare to a westernised or typical Australian diet? *Similar/Different*

Before your child was suspected of having IBD,

- did the child's **usual diet** differ from **traditional diet** (i.e., non-western) **described above**? *Yes/No*

(Applicable if 1 or both parents select non-Caucasian ethnic background)

Before your child was suspected of having IBD: **(Applicable if 1 or both parents select non-Caucasian ethnic background)*

	never	sometimes	often	very often
how often did your child eat typical **Australian food for breakfast** (*such as cereal/toast/porridge etc.*) *				
how often did your child eat typical **Australian food for lunch** (*such as sandwich/burgers etc.*) *				
how often did your child eat typical **Australian food for dinner** (*such as meat/chicken and vegetables/spaghetti bolognaise etc.*) *				
how often did your child eat typical **Australian food for snack** (*such as chips/crackers/muesli bars/biscuits etc.*) *				
how often did your child eat typical **Australian food for dessert** (*such as cakes/puddings/pastries etc.*) *				
how often did your child eat **your traditional food for breakfast***				
how often did your child eat **your traditional food for lunch***				
how often did your child eat **your traditional food for dinner***				
how often did your child eat **your traditional food for snack***				
how often did your child eat **your traditional food for dessert***				

Before your child was suspected of having IBD, **(Applicable if 1 or both parents select non-Caucasian ethnic background)*

-did mother's **usual diet** differ from your **traditional diet described above**? * *Yes/No*

If yes, how:

-did father's **usual diet** differ from your **traditional diet described above**? * *Yes/No*

If yes, how:

Before your child was suspected of having IBD, how often did you use **convenience** food items for **home-prepared meals** *(e.g., Curry pastes, frozen/re-heatable meals)*?

Never/Rarely *1-3/month* *1-2/week* *3-5/week* *1/day* *>2/day*

Before your child was suspected of having IBD, how often did you use **basic food groups** *(e.g., grains, vegetables, meat/chicken/fish/eggs, legumes/lentils/nuts & seeds)* **in home-prepared meals**?

Never/Rarely *1-3/month* *1-2/week* *3-5/week* *1/day* *>2/day*

Before your child was suspected of having IBD, how many **dine-in/takeaway meals** did your child usually have? *Never/Rarely* *1-3/month* *1-2/week* *3-5/week* *1/day* *>2/day*

How often would the dine-in/takeaway meals be of the **fast-food** type (*e.g., chain type food outlets*)?

50% of the time / *<50% of the time* / *>50% of the time*

<u>Before</u> your child was suspected of having IBD, which ***type of oil/fat*** did you use routinely in cooking/food preparation?
If your child was ever **breastfed**:
What age your child **stopped receiving any breast milk**? *months/year*
What age your child first had **any food or drink other than breast milk** (including water, formula and juice)? *months/year*
What age your child first **ate any soft, semi-solid or solid food**? *months/year*

<u>Before</u> your child was suspected of having IBD, how **often** did they have the following **foods/drinks?**

	Never	*6-9 times/year*	*1-3/month*	*1-2/week*	*3-6/week*	*1/day*
Vegetables						
Fruits						
Legumes/Lentils						
Nuts/Seeds						
Rice						
Wholegrain/wholemeal bread/wholemeal roti						
Other refined grains (e.g., white bread)						
Milk/Cheese (plain)						
Yoghurt (plain)						
Turmeric						
Other Added herbs/spice						
Fermented foods (e.g., Kefir, Kimchi, Sauerkraut, Dosa/Idli)						
Juice						
Soft Drink/Carbonated drinks						
Flavoured Milk/Milk products (incl yoghurts)						
Processed (Deli) meats						
Red meat						
Store-bought Frozen dairy dessert/Ice-cream						
Store-bought Savoury snacks/biscuits						
Butter/Ghee/Lard						
Coconut oil						
Added sugars (sugar, honey, syrup, palm sugar)						

<u>Before</u> your child was suspected of having IBD, **how many different number of plant-based foods** did they consume in a <u>typical/usual week</u> *(includes, Vegetables, Fruits, Cereals/Grains, Legumes and lentils, Nuts and seeds -all of these in unprocessed/minimally processed form)*? ____

<u>Before</u> your child was suspected of having IBD, did you make **changes in their diet** to help ease their gastrointestinal symptoms? Yes/No
If **yes,** what were the changes made in your child's diet to help ease their IBD symptoms?
If **yes,** were the dietary changes **guided by an Accredited Practising Dietitian**? *Yes/No/unsure*
Are there any foods that you believe to **trigger** your child's IBD symptoms?
Do you have any food and nutrition concerns? *Yes/No*
Comments:
Have you sought **Complementary and Alternative Medicine** therapy for your child to help with IBD type symptoms? *Yes/No*

If **yes,** please provide details on the type/form (how much and how often):

<u>Environmental - Physical Activity Levels</u>
how often did they engage in an **outdoor physical activity (daytime)** like sports/walking/bike riding/playing outdoors etc.?
Never/Rarely 1-3/month 1-2/week 3-5/week 1/day ≥2/day

Comments: *<u>duration</u> of daytime outdoor physical activity for the frequency selected above?*

<u>Growth History (</u>Z scores of weight, height and BMI):

<u>Before</u> your child was suspected of having IBD, did you notice any **<u>unintentional</u> weight loss**? *Yes/No*
If yes, how much *(provide details):____ kg* *and over what period? ____ months /____ years*

Comments:

<u>Environmental-Other factors</u>
<u>Before</u> your child was suspected of having IBD, did your family have any **pet/s** in your home?
Yes / No / Unsure (If yes, please tick below on the sort of pet)
Dog
Cat
Birds
Rodents
Aquarium
Other
Comments:

<u>Before</u> your child was suspected of having IBD, did your home have a **vegetable garden**?
Yes / No / Unsure
Comments:

Smoking exposure:
Mother's smoking history: *Yes / Ex/ Never/ Unsure*
If yes/ex, were you smoking when you were pregnant with the child who has been suspected of having IBD?
If yes, provide details: number of cigarettes used/day: only socially:

Father's smoking history: *Yes / Ex/ Never/ Unsure*
If yes/ex, were you smoking when your wife/partner was pregnant with the child who has been suspected of having IBD?
If yes, provide details: number of cigarettes used/day: only socially:

Child's smoking history:
Active exposure: *Yes / Ex/ Never/ Unsure.* **If yes**, provide details: number of cigarettes used/day: only socially:
Passive exposure by parents/carer: *Yes / Ex/ Never/ Unsure*
Did <u>any other people</u> who lived in your home with your child, smoke in your home?
Yes / Ex/ Never/ Unsure
Comments:

How **long** has your child lived in Australia? <5years >5 to 10 years >10 years

How **long** have you (i.e., parents/caregivers) lived in Australia? <5years >5 to 10 years >10 years

Does any **older relative** live with the child in the house? *Yes/No*

*(Applicable if 1 or both parents select non-Caucasian ethnic background)	never	sometimes	often	very often
How often does your child **speak** with you and your family in **English**? *				
How often does your child **speak** with his/her friends in **English**? *				
How often does your child **speak** with you and your family in **your regional language** (i.e., heritage/ethnic language)? *				
How often does your child **speak** with his/her friends in **your regional language** (i.e., heritage/ethnic language)? *				

Please list details of all the **other countries** that the child has ever lived and approximate duration at each of these (Apart from country of birth and Australia)

Other countries lived *Dates (start and end date)* *Duration*

Please list details of the **Suburb/Postcodes (within Australia)** for all the places the child has ever lived and approximate duration at each of these

Other suburbs/postcodes lived *Dates (start and end date)* *Duration*

What is the total annual income of your household (including all income earning household members)**?**
Income includes: wages and salaries; government pensions, benefits and allowances; profit or losses from business or investments; other sources of income. Do not deduct: Tax, superannuation contributions, amounts salary sacrificed, or any other automatic deductions

Nil income *Negative income* *Don't know* *Don't want to answer*

$3,000 or more per week ($156,000 or more per year)

$2,000 - $2,999 per week ($104,000 - $155,999 per year)

$1,750 - $1,999 per week ($91,000 - $103,999 per year)

$1,500 - $1,749 per week ($78,000 - $90,999 per year)

$1,250 - $1,499 per week ($65,000 - $77,999 per year)

$1,000 - $1,249 per week ($52,000 - $64,999 per year)

$800 - $999 per week ($41,600 - $51,999 per year)

$650 - $799 per week ($33,800 - $41,599 per year)

$500 - $649 per week ($26,000 - $33,799 per year)

$400 - $499 per week ($20,800 - $25,999 per year)

$300 - $399 per week ($15,600 - $20,799 per year)

$150 - $299 per week ($7,800 - $15,599 per year)

$1 - $149 per week ($1 - $7,799 per year)

FUNCTIONING AND WELLBEING

Days missed from school in the last 6 months related to symptoms from being suspected of having IBD:

Does your child currently have a flare of suspected IBD type symptoms? *Yes/No / Unsure*

Notes:

PLEASE NOTE: This form will be adapted for REDCAP like all other tools used in this study and skip logic will be used so only relevant questions are asked.
