# Supplementary material for: Pre-Diagnosis Dietary Pattern Differences in Australian Children with Inflammatory Bowel Disease: Exposure Across Ethnicities
Source: Nutrients. 2026 Apr 22;18(9):1313. doi: 10.3390/nu18091313 (PMC13165386; doi:10.3390/nu18091313)
Supplement: Supplementary file 1 [file nutrients-18-01313-s001.zip › Table S3 Pre-diagnosis food frequency.docx]

**Supplementary Table** S3 presents frequencies of pre-diagnosis intake of whole foods and discretionary foods. This has been presented by IBD subtype (CD vs UC) and ethnicity subtype (Caucasian vs. non-Caucasian)

**Supplementary Table** S3: **Pre-diagnosis reported exposure to whole foods and discretionary foods**

|  | **IBD**  **n (%)** | **Ulcerative Colitis**  **n (%)** | **Crohn's Disease**  **n (%)** | **P**  **value** | **Caucasian**  **n (%)** | **Non-Caucasian**  **n (%)** | **P value** |
| --- | --- | --- | --- | --- | --- | --- | --- |
|  | N = 51 | **22 (43.1%)** | **29 (56.8%)** |  | **28**  **(54.9%)** | **23**  **(45%)** |  |
| Vegetables |  |  |  | **0.012*** |  |  | 0.49 |
| *Frequent Intake* >/=3 days/week | 39 (76) | 14 (64%) | 25 (86%) |  | 23 (82) | 16 (70) |  |
| 1-2 days/week | 7 (14) | 6 (27.2) | 1 (3) |  | 2 (7) | 5 (22) |  |
| 1-3/month | 2 (4) | 2 (9) | 0 |  | 1 (4) | 1 (4) |  |
| Never | 3 (6) | 0 | 3 (10) |  | 2 (7) | 1 (4) |  |
| Fruits |  |  |  | 0.28 |  |  | 0.45 |
| *Frequent Intake* >/=3 days/week | 42 (82) | 18 (82%) | 24 (83%) |  | 22 (79) | 20 (87) |  |
| 1-2 days/week | 4 (8) | 2 (9) | 2 (7) |  | 3 (11) | 1 (4) |  |
| 1-3/month | 2 (4) | 2 (9) | 0 |  | 1 (3) | 1 (4) |  |
| 6-9/year | 1 (2) | 0 | 1 (3) |  | 0 | 1 (4) |  |
| Never | 2 (4) | 0 | 2 (7) |  | 2 (7) | 0 |  |
| Dairy-Unflavoured Milk/Cheese |  |  |  | 0.24 |  |  | 0.97 |
| *Frequent Intake* >/=3 days/week | 35 (68) | 15 (68%) | 20 (69%) |  | 20 (71) | 15 (65.2) |  |
| 1-2 days/week | 7 (14) | 5 (23) | 2 (7) |  | 3 (11) | 4 (17.3) |  |
| 1-3/month | 2 (4) | 1 (4.5) | 1 (3) |  | 1 (4) | 1 (4.3) |  |
| Never | 7(14) | 1 (4.5) | 6 (21) |  | 4 (14) | 3 (13) |  |
| Grain based foods |  |  |  |  |  |  |  |
| a)White Rice |  |  |  | 0.44 |  |  | **0.009*** |
| *Frequent Intake* >/=3 days/week | 18 (35) | 8 (36) | 10 (35) |  | 5 (18) | 13 (57) |  |
| 1-2 days/week | 23 (45) | 10 (45.4) | 13 (45) |  | 13 (46) | 10 (43) |  |
| 1-3/month | 6 (12) | 3 (14) | 3 (10) |  | 6 (21) | 0 |  |
| 6-9/year | 1 (2) | 1 (4.5) | 0 |  | 1 (4) | 0 |  |
| Never | 3 (6) | 0 | 3 (10) |  | 3 (11) | 0 |  |
| *Infrequent/Never Intake:* </=1-2 days/week i.e. 1-2 days/week, 1-3/month , 6-9/year and never, are all combined | 33 (65) | 14 (64) | 19 (65) |  | 10 (82) | 10 (43) |  |
| b) Wholemeal/Wholegrain Bread |  |  |  | 0.82 |  |  | 0.34 |
| *Frequent Intake* >/=3 days/week | 20 (39) | 7 (32) | 13 (45) |  | 12 (43) | 8 (35) |  |
| 1-2 days/week | 12 (23.5) | 6 (27) | 6 (21) |  | 4 (14) | 8 (35) |  |
| 1-3/month | 4 (8) | 2 (9) | 2 (7) |  | 2 (7) | 2 (8) |  |
| 6-9/year | 0 | 0 | 0 |  | 0 | 0 |  |
| Never | 15 (29.4) | 7 (32) | 8 (27) |  | 10 (36) | 5 (22) |  |
| *Infrequent/Never* Intake: </=1-2 days/week i.e. 1-2 days/week, 1-3/month , 6-9/year and never, are all combined | 31 (61) | 68% | 55% |  | 57% | 65% |  |
| c) Other Refined Grain Products (e.g. white bread) |  |  |  | 0.24 |  |  | 0.78 |
| *Frequent Intake* >/=3 days/week | 26 (51) | 12 (55) | 14 (48) |  | 13 (46) | 13 (56) |  |
| 1-2 days/week | 10 (19.6) | 6 (27) | 4 (14) |  | 6 (21) | 4 (17) |  |
| 1-3/month | 5 (10) | 1 (4.5) | 4 (14) |  | 3 (11) | 2 (9) |  |
| 6-9/year | 3 (6) | 2 (9) | 1 (3) |  | 1 (4) | 2(9) |  |
| Never | 7 (14) | 1 (4.5) | 6 (21) |  | 5 (18) | 2 (9) |  |
| Lentils/Legumes |  |  |  | 0.32 |  |  | 0.11 |
| *Frequent Intake* >/=3 days/week | 6 (12) | 3 (14) | 3 (10) |  | 1 (3.5) | 5 (22) |  |
| 1-2 days/week | 12 (23.5) | 6 (27) | 6 (21) |  | 5 (18) | 7 (30) |  |
| 1-3/month | 11 (21.5) | 7 (32) | 4 (14) |  | 6 (21) | 5 (22) |  |
| 6-9/year | 9 (18) | 2 (9) | 7 (24) |  | 7 (25) | 2(9) |  |
| Never | 13 (25) | 4 (18) | 9 (31) |  | 9 (32) | 4 (17) |  |
| *Less often /Never:* 1-3/month , 6-9/year and never, are all combined | 33 (65) | 13 (59) | 20 (69) |  | 79% | 48% |  |
| Nuts/Seeds |  |  |  | 0.66 |  |  | 0.08 |
| *Frequent Intake* >/=3 days/week | 6 (12) | 3 (14) | 3 (10) |  | 5 (18) | 1 (4) |  |
| 1-2 days/week | 14 (27) | 4 (18%) | 10 (34) |  | 8 (29) | 6 (26) |  |
| 1-3/month | 12 (23.5) | 6 (27) | 6 (21) |  | 3 (11) | 9 (39) |  |
| 6-9/year | 6 (12) | 2 (9) | 4 (14) |  | 5 (18) | 1 (4) |  |
| Never | 13 (25.4) | 7 (32) | 6 (21) |  | 7 (25) | 6 (26) |  |
| *Less often /Never:* 1-3/month , 6-9/year and never, are all combined | 31(61) | 68% | 56% |  | 54% | 69% |  |
| Turmeric |  |  |  | 0.22 |  |  | 0.01 |
| *Frequent Intake* >/=3 days/week | 7 (13) | 3 (13.5) | 4 (14) |  | 0 | 7 (30) |  |
| 1-2 days/week | 3 (6) | 3 (14) | 0 |  | 0 | 3 (13) |  |
| 1-3/month | 6 (12) | 1 (4.5) | 5 (17) |  | 4 (14.2) | 2 (9) |  |
| 6-9/year | 9 (18) | 4 (18) | 5 (17) |  | 6 (21.4) | 3 (13) |  |
| Never | 26 (51) | 11 (50) | 15 (52) |  | 18 (64.2) | 8 (35) |  |
| Added Herbs and Spices |  |  |  | 0.45 |  |  | 0.15 |
| *Frequent Intake* >/=3 days/week | 24 (47) | 8 (36) | 16 (55) |  | 9 (32) | 15 (65) |  |
| 1-2 days/week | 16 (31) | 7 (32) | 9 (31) |  | 13 (46) | 3 (13) |  |
| 1-3/month | 3 (6) | 2 (9) | 1 (3.4) |  | 2 (7) | 1 (4.3) |  |
| 6-9/year | 2 (4) | 2 (9) | 0 |  | 1 (4) | 1 (4.3) |  |
| Never | 6 (12) | 3 (14) | 3 (10.3) |  | 3(11) | 3 (13) |  |
| *Infrequent/Never* Intake: </=1-2 days/week i.e. 1-2 days/week, 1-3/month , 6-9/year and never, are all combined | 27 (53) | 14 (64) | 13 (45) |  | 19 (68) | 8 (35) |  |
| Fermented foods |  |  |  | 0.19 |  |  | 0.19 |
| *Frequent Intake* >/=3 days/week | 4 (7) | 2 (9) | 2 (7) |  | 1 (3) | 3 (13) |  |
| 1-2 days/week | 5 (10) | 1 (4.5) | 4 (14) |  | 2 (7) | 3 (13) |  |
| 1-3/month | 3 (6) | 0 | 3 (10.3) |  | 3 (11) | 0 |  |
| 6-9/year | 2 (4) | 0 | 2 (7) |  | 0 | 2 (9) |  |
| Never | 37 (73) | 19 (86.3) | 18 (62) |  | 22 (79) | 15 (65) |  |
| Ingredients/foods of concern in the context of IBD | | | | | | | |
| Red Meat |  |  |  | 0.09 |  |  | 0.10 |
| *Regular Intake* >/=1-2 days/week | 39 (76) | 16 (73) | 23 (79.3) |  | 22 (78) | 17 (74) |  |
| 1-3/month | 3 (6) | 2 (9) | 1 (3.4) |  | 0 | 3 (13) |  |
| 6-9/year | 3 (6) | 3 (14) | 0 |  | 3 (11) | 0 |  |
| Never | 6 (12) | 1 (4) | 5 (17.2) |  | 3 (11) | 3 (13) |  |
| Processed/deli meat |  |  |  | 0.41 |  |  | **0.02*** |
| *Frequent Intake* >/=3 days/week | 20 (39) | 7 (32) | 13 (45) |  | 15 (54) | 5 (22) |  |
| 1-2 days/week | 9 (18) | 5 (23) | 4 (14) |  | 5 (18) | 4 (17) |  |
| 1-3/month | 11 (22) | 4 (18) | 7 (24) |  | 4 (14) | 7 (30.4) |  |
| 6-9/year | 2 (4) | 2 (9) | 0 |  | 2 (7) | 0 |  |
| Never | 9 (17) | 4 (18) | 5 (17) |  | 2 (7) | 7 (30.4) |  |
| *Regular Intake* >/=1-2 days/week  (*Regular Intake: includes* 1-2 days/week and >/=3 days/week) | 29 (57) | 12 (55) | 17 (59) |  | 20 (72) | 9 (39) |  |
| Saturated fat |  |  |  | 0.22 |  |  | 0.25 |
| *Regular Intake* >/=1-2 days/week | 35 (70) | 14 (64) | 21(75) |  | 18 (67) | 17 (74) |  |
| 1-3/month | 6 (12) | 5 (23) | 1 (4) |  | 4 (15) | 2 (9) |  |
| 6-9/year | 3 (6) | 1 (4) | 2 (7) |  | 3 (11) | 0 |  |
| Never | 6 (12) | 2 (9) | 4 (14) |  | 2 (7) | 4 (17) |  |
| Store-bought frozen dairy dessert |  |  |  | 0.70 |  |  | 0.43 |
| *Regular Intake* >/=1-2 days/week | 35 (69) | 16 (73) | 19 (66) |  | 21 (75) | 14 (61) |  |
| 1-3/month | 11 (21) | 5 (23) | 6 (21) |  | 4 (14) | 7 (30) |  |
| 6-9/year | 4 (8) | 1 (4) | 3 (10) |  | 2 (7) | 2 (9) |  |
| Never | 1 (2) | 0 | 1 (3) |  | 1 (4) | 0 |  |
| Store-bought savoury snacks/ biscuits |  |  |  | 0.44 |  |  | 0.42 |
| *Regular Intake* >/=1-2 days/week | 47 (92) | 21 (95) | 26 (90) |  | 25 (89) | 22 (96) |  |
| 1-3/month | 2 (4) | 1 (5) | 1 (3) |  | 2 (7) | 0 |  |
| 6-9/year | 2 (4) | 0 | 2 (7) |  | 1 (4) | 1 (4) |  |
| Never | 0 | 0 | 0 |  | 0 | 0 |  |
| Soft drinks |  |  |  | 0.62 |  |  | 0.44 |
| *Regular Intake* >/=1-2 days/week | 25 (49) | 13 (59) | 12 (41.3) |  | 12 (43) | 13 (57) |  |
| 1-3/month | 7 (14) | 2 (9) | 5 (17.2) |  | 3 (11) | 4 (17) |  |
| 6-9/year | 5 (10) | 2 (9) | 3 (10.3) |  | 4 (14) | 1 (4) |  |
| Never | 14 (27) | 5 (23) | 9 (31) |  | 9 (32) | 5 (22) |  |
| Fruit juice |  |  |  | 0.40 |  |  | 0.44 |
| *Regular Intake* >/=1-2 days/week | 29 (57) | 13 (59) | 16 (55) |  | 14 (50) | 15 (65) |  |
| 1-3/month | 10 (20) | 6 (27) | 4 (14) |  | 5 (18) | 5 (22) |  |
| 6-9/year | 5 (10) | 1 (5) | 4 (14) |  | 4 (14) | 1 (4) |  |
| Never | 7 (14) | 2 (9) | 5 (17) |  | 5 (18) | 2 (9) |  |
| Dairy-Flavoured Milk/Yoghurt |  |  |  | **0.04*** |  |  | 0.46 |
| *Regular Intake* >/=1-2 days/week | 30 (58.5) | 11 (50) | 19 (66) |  | 17 (61) | 13 (57) |  |
| 1-3/month | 6 (12) | 5 (23) | 1 (3) |  | 4 (14) | 2 (9) |  |
| 6-9/year | 4 (8) | 0 | 4 (14) |  | 3 (11) | 1 (4) |  |
| Never | 11 (21.5) | 6 (27) | 5 (17) |  | 4 (14) | 7 (30) |  |
| Dining out/having takeaway meals —  *Regular Intake* >/=1-2 days/week | 32 (63) | 11 (50) | 29 (72) | 0.23 | 17 (61) | 15 (65) | 0.65 |
| Fast foods intake >/= 50% of time | 29 (58) | 14 (64) | 15 (54) | 0.46 | 15 (56) | 14 (61) | 0.40 |

**Study title:** **Pre-Diagnosis Dietary Pattern Differences in Australian Children with Inflammatory Bowel Disease: Exposure Across Ethnicities**

Nisha Thacker^1,2^ **M. Nutr. & Diet**.[Nisha.Thacker@uon.edu.au](mailto:Nisha.Thacker@uon.edu.au)

Shoma Dutt^3,4^ **PhD** [shoma.dutt@health.nsw.gov.au](mailto:shoma.dutt@health.nsw.gov.au)

Emily C. Hoedt^5,6^ **PhD** [Emily.Hoedt@newcastle.edu.au](mailto:Emily.Hoedt@newcastle.edu.au)

Edward V O’Loughlin^3^ **MD** [ted.oloughlin@health.nsw.gov.au](mailto:ted.oloughlin@health.nsw.gov.au)

Clare E Collins^1,2^ **PhD** [clare.collins@newcastle.edu.au](mailto:clare.collins@newcastle.edu.au)

Kerith Duncanson^2,5,7^ **PhD** [kerith.duncanson@newcastle.edu.au](mailto:kerith.duncanson@newcastle.edu.au) (corresponding author)

The Children’s Hospital Westmead, Sydney Children’s Hospital Network, Australia
