# Supplementary material for: Pre-Diagnosis Dietary Pattern Differences in Australian Children with Inflammatory Bowel Disease: Exposure Across Ethnicities
Source: Nutrients. 2026 Apr 22;18(9):1313. doi: 10.3390/nu18091313 (PMC13165386; doi:10.3390/nu18091313)
Supplement: Supplementary file 1 [file nutrients-18-01313-s001.zip › Table S4 Association between number of different plant foods consumed per week a) Paediatric IBD subtype (t-test) and b) Ethnicity (t-test).docx]

**Supplementary Table S4:** **Association between number of different plant foods consumed per week : a) Paediatric IBD subtype (t-test) and b) Ethnicity (t-test)**

| **a)Two-sample t test with unequal variances: number of different plant foods intake per week and paediatric IBD subtype** | | | | | | |
| --- | --- | --- | --- | --- | --- | --- |
| **Group** | **N** | **Mean** | **Std. err.** | **Std. dev.** | **95% CI**  **(lower)** | **95% CI**  **(upper)** |
| **Paediatric IBD subtype** |  |  |  |  |  |  |
| **UC** | 22 | 10.72 | 1.09 | 5.15 | 8.44 | 13.01 |
| **CD** | 29 | 12.20 | 1.04 | 5.60 | 10.07 | 14.33 |
| Combined | 51 | 11.56 | 0.75 | 5.41 | 10.04 | 13.09 |
| diff |  | -1.47 | 1.51 |  | -4.52 | 1.56 |
| diff = mean (0) - mean (1) t = -0.9776  H0: diff = 0 Satterthwaite's degrees of freedom = 47.1141  Ha: diff < 0 Ha: diff! = 0 Ha: diff > 0  Pr (T < t) = 0.1666 Pr (\|T\| > \|t\|) = 0.3333 Pr (T > t) = 0.8334 | | | | | | |
| **b)Two-sample t test with unequal variances: number of different plant foods intake per week and ethnicity** | | | | | | |
| **Ethnicity** | **N** | **Mean** | **Std. err.** | **Std. dev.** |  |  |
| **Non-Caucasian** | 23 | 11.91 | 1.18 | 5.68 | 9.45 | 14.36 |
| **Caucasian** | 28 | 11.28 | 0.99 | 5.26 | 9.24 | 13.32 |
| Combined | 51 | 11.56 | 0.75 | 5.41 | 10.04 | 13.09 |
| diff |  | 0.62 | 1.54 |  | -2.48 | 3.74 |
| diff = mean(0) - mean(1) t = 0.4054  H0: diff = 0 Satterthwaite's degrees of freedom = 45.5508  Ha: diff < 0 Ha: diff != 0 Ha: diff > 0  Pr(T < t) = 0.6565 Pr(\|T\| > \|t\|) = 0.6871 Pr(T > t) = 0.3435 | | | | | | |

CI = confidence interval
