# Supplementary material for: Pre-Diagnosis Dietary Pattern Differences in Australian Children with Inflammatory Bowel Disease: Exposure Across Ethnicities
Source: Nutrients. 2026 Apr 22;18(9):1313. doi: 10.3390/nu18091313 (PMC13165386; doi:10.3390/nu18091313)
Supplement: Supplementary file 1 [file nutrients-18-01313-s001.zip › Table S5 Number of different plant foods intake per week based on paediatric IBD subtype and ethnicity.docx]

**Supplementary File S5: Number of different plant foods intake per week based on paediatric IBD subtype and ethnicity**

| Total number of different plant foods intake per week by IBD subtype | | | | | | |
| --- | --- | --- | --- | --- | --- | --- |
| IBD subtype | **Mean** | **SD** | **Median(p50)** | **p25** | **p75** | **N** |
| UC | 10.72 | 5.15 | 9.5 | 7 | 15 | 22 |
| CD | 12.20 | 5.60 | 12 | 8 | 16 | 29 |
| IBD | 11.56 | 5.41 | 11 | 8 | 16 | 51 |
| Total number of different plant foods intake per week by ethnicity | | | | | | |
| Non-Caucasian | 11.91 | 5.68 | 10 | 8 | 16 | 23 |
| Caucasian | 11.28 | 5.26 | 12 | 8 | 15.5 | 28 |
| Total | 11.56 | 5.41 | 11 | 8 | 16 | 51 |
| Total number of different plant foods intake per week by ethnicity sub-groups | | | | | | |
| Caucasian | 11.28 | 5.26 | 12 | 8 | 15.5 | 28 |
| Indigenous | 13 | - | 13 | 13 | 13 | 1 |
| South Asian | 16.4 | 7.16 | 18 | 15 | 20 | 5 |
| Middle Eastern | 9.33 | 5.22 | 8 | 8 | 10 | 9 |
| Asian | 10.33 | 1.15 | 11 | 9 | 11 | 3 |
| Other | 12 | - | 12 | 12 | 12 | 1 |
| Mixed Ethnicity | 13 | 6.05 | 12.5 | 8 | 18 | 4 |
| Total | 11.56 | 5.41 | 11 | 8 | 16 | 51 |

**Study title:** **Pre-Diagnosis Dietary Pattern Differences in Australian Children with Inflammatory Bowel Disease: Exposure Across Ethnicities**

Nisha Thacker^1,2^ **M. Nutr. & Diet**.[Nisha.Thacker@uon.edu.au](mailto:Nisha.Thacker@uon.edu.au)

Shoma Dutt^3,4^ **PhD** [shoma.dutt@health.nsw.gov.au](mailto:shoma.dutt@health.nsw.gov.au)

Emily C. Hoedt^5,6^ **PhD** [Emily.Hoedt@newcastle.edu.au](mailto:Emily.Hoedt@newcastle.edu.au)

Edward V O’Loughlin^3^ **MD** [ted.oloughlin@health.nsw.gov.au](mailto:ted.oloughlin@health.nsw.gov.au)

Clare E Collins^1,2^ **PhD** [clare.collins@newcastle.edu.au](mailto:clare.collins@newcastle.edu.au)

Kerith Duncanson^2,5,7^ **PhD** [kerith.duncanson@newcastle.edu.au](mailto:kerith.duncanson@newcastle.edu.au) (corresponding author)

The Children’s Hospital Westmead, Sydney Children’s Hospital Network, Australia
