# Supplementary material for: Pre-Diagnosis Dietary Pattern Differences in Australian Children with Inflammatory Bowel Disease: Exposure Across Ethnicities
Source: Nutrients. 2026 Apr 22;18(9):1313. doi: 10.3390/nu18091313 (PMC13165386; doi:10.3390/nu18091313)
Supplement: Supplementary file 1 [file nutrients-18-01313-s001.zip › Table S6 Clinical characteristics.docx]

**Supplementary Table S6:** **Clinical characteristics**

|  | **Caucasian** | **Non-Caucasian** | **P**  **value** | **Ulcerative Colitis** | **Crohn's Disease** | **P**  **value** |
| --- | --- | --- | --- | --- | --- | --- |
| N = 56 | **N=31**  **(55.4%)** | **N=25**  **(44.6%)** |  | **N=26**  **(46.4%)** | **N=30**  **(53.6%)** |  |
| Endoscopic Severity Score: Crohn’s Disease (mean+SD) | N=11 | N=7 | 0.48 | n/a | 15.7+9.59  N=18 |  |
| Mild (^c^SES 3-6) | 1 (9.09%) | 0 |  | n/a | 1 (5.5%) |  |
| Moderate (^c^SES 7-15) | 5 (45.4%) | 5 (71.4%) |  | n/a | 9 (50%) |  |
| Severe (^c^SES >15) | 5 (45.4%) | 2 (28.5%) |  | n/a | 8 (44.4%) |  |
| Endoscopic Severity Score: Ulcerative colitis - Mayo Score (mean+SD) | N=11 | N=12 | 0.24 | 2.3+0.65  N=23 | n/a |  |
| Mild (Mayo score 1) | 2 (18.1%) | 0 |  | 2 (8.7%) |  |  |
| Moderate (Mayo score 2) | 5 (45.4%) | 5 (41.6%) |  | 10  (43.4%) |  |  |
| Severe (Mayo score 3) | 4 (36.3%) | 7 (58.3%) |  | 11  (47.8%) |  |  |
| Crohn’s Disease Phenotype: Paris classification (N = 23) |  |  |  | n/a |  |  |
| Age at Diagnosis |  |  | 0.17 |  |  |  |
| A1a (< 10 years) | 3 (20%) | 0 |  |  | 3 (13.04%) |  |
| A1b (10-17 years) | 12 (80%) | 8 (100%) |  |  | 20 (86.96%) |  |
| Location L1: Distal 1/3 ileum +limited caecal disease | 2 (13.3%) | 1 (12.5%) | 0.95 |  | 3 (13.04%) |  |
| Location L2: Colonic | 5 (33.3%) | 3  (37.5%) | 0.84 |  | 8 (34.78%) |  |
| Location L3: Ileocolonic | 9 (60%) | 4 (50%) | 0.64 |  | 13 (56.52%) |  |
| Location L4a: Upper disease proximal to Ligament of Treitz | 3 (20%) | 2 (25%) | 0.78 |  | 5 (21.74%) |  |
| Location L4b: Upper disease distal to Ligament of Treitz and proximal to distal 1/3 ileum | 2 (13.3%) | 0 | 0.28 |  | 2 (8.7%) |  |
| Behaviour B1: Non stricturing & non penetrating | 12 (80%) | 8 (100%) | 0.17 |  | 20 (86.96%) |  |
| Behaviour B2: Stricturing | 1 (6.6%) | 0 | 0.45 |  | 1 (4.35) |  |
| Behaviour B3: Penetrating | 1 (6.6%) | 0 | 0.45 |  | 1 (4.35%) |  |
| Behaviour_B2B3: Both, stricturing & penetrating disease | 0 | 0 | n/a |  | None |  |
| Behaviour P: Perianal disease modifier | 3 (20%) | 1(12.5%) | 0.65 |  | 4 (17.39%) |  |
| Growth |  |  | 0.78 |  |  |  |
| G_0_ = No evidence of growth delay | 12 (80%) | 6 (75%) |  |  | 18 (78.26%) |  |
| G_1_ = Growth delay | 3 (20%) | 2 (25%) |  |  | 5 (21.74%) |  |
| Ulcerative Colitis Phenotype: Paris classification (N = 24) |  |  |  |  | n/a |  |
| Extent |  |  |  |  |  |  |
| E 1: Ulcerative Proctitis | 0 | 0 | n/a | None |  |  |
| E2: Left Sided ulcerative colitis (distal to splenic flexure) | 2 (18.1%) | 1  (7.6%) | 0.43 | 3 (12.50%) |  |  |
| E3: Extensive (hepatic flexure distally) | 0 | 3 (23.08%) | 0.08 | 3 (12.50%) |  |  |
| E4: Pancolitis (Proximal to hepatic flexure) | 9 (81.8%) | 9  (69.2%) | 0.47 | 18 (75%) |  |  |
| Severity |  |  |  |  |  |  |
| S0: never severe^#^ | 4 (36.3%) | 4  (30.7%) | 0.77 | 8 (33.33%) |  |  |
| S1: ever severe^#^ | 2 (18.1%) | 3  (23.08%) | 0.76 | 5 (20.83%) |  |  |
| Physician Global Assessment: |  |  |  |  |  |  |
| Mild | 5 (20%) | 3 (18.7%) | 0.97 | 3 (16.7%) | 5 (21.7%) | 0.16 |
| Moderate | 16 (64%) | 10 62.5%) |  | 14 (77.8%) | 12(52.2%) |  |
| Severe | 4 (16%) | 3 (18.7%) |  | 1 (5.6%) | 6 (26.1%) |  |

^c^ SES: Simple Endoscopic Score**; ^#^** Severe defined by ^d^PUCAI > or = 65; ^d^ PUCAI Paediatric Ulcerative Colitis Activity Index

**Note:** For some variables, data were not available for all participants; therefore, sample sizes (N) are smaller than the total study population

**Study title:** **Pre-Diagnosis Dietary Pattern Differences in Australian Children with Inflammatory Bowel Disease: Exposure Across Ethnicities**

Nisha Thacker^1,2^ **M. Nutr. & Diet**.[Nisha.Thacker@uon.edu.au](mailto:Nisha.Thacker@uon.edu.au)

Shoma Dutt^3,4^ **PhD** [shoma.dutt@health.nsw.gov.au](mailto:shoma.dutt@health.nsw.gov.au)

Emily C. Hoedt^5,6^ **PhD** [Emily.Hoedt@newcastle.edu.au](mailto:Emily.Hoedt@newcastle.edu.au)

Edward V O’Loughlin^3^ **MD** [ted.oloughlin@health.nsw.gov.au](mailto:ted.oloughlin@health.nsw.gov.au)

Clare E Collins^1,2^ **PhD** [clare.collins@newcastle.edu.au](mailto:clare.collins@newcastle.edu.au)

Kerith Duncanson^2,5,7^ **PhD** [kerith.duncanson@newcastle.edu.au](mailto:kerith.duncanson@newcastle.edu.au) (corresponding author)

The Children’s Hospital Westmead, Sydney Children’s Hospital Network, Australia
