# Supplementary material for: Pre-Diagnosis Dietary Pattern Differences in Australian Children with Inflammatory Bowel Disease: Exposure Across Ethnicities
Source: Nutrients. 2026 Apr 22;18(9):1313. doi: 10.3390/nu18091313 (PMC13165386; doi:10.3390/nu18091313)
Supplement: Supplementary file 1 [file nutrients-18-01313-s001.zip › Table S7 Dietary Acculturation factors..docx]

**Supplementary File S7:** Dietary Acculturation factors-Child

**Table S7.1** Child's usual pre-diagnosis diet and traditional influence (n=23)

|  | CD | UC | PIBD |
| --- | --- | --- | --- |
| N= 23 |  |  |  |
| Usual pre-diagnosis diet did **not** differ from the traditional diet | 36.36% | 58.33% | 47.83% |
| Usual pre-diagnosis diet differed from the traditional diet | 63.64% | 41.67% | 52.17% |

**Figure S7.2** Child's usual pre-diagnosis traditional **breakfast** choice (n=23)

**Figure S7.3** Child's usual pre-diagnosis traditional **lunch** choice (n=23)

**Figure S7.4** Child's usual pre-diagnosis traditional **dinner** choice (n=23)

**Figure S7.5** Child's usual pre-diagnosis traditional **snack** choice (n=23)

**Figure S7.6** Child's usual pre-diagnosis traditional **dessert** choice (n=23)

**Figure S7.7** Dietary acculturation amongst parents

1. Before Child's IBD diagnosis, did mother's diet differ from the traditional diet

|  | CD | UC | PIBD |
| --- | --- | --- | --- |
| N= 22 |  |  |  |
| Before Child's IBD diagnosis , mother’s usual diet did **not** differ from the traditional diet | 30.00% | 58.33% | 45.45% |
| Before Child's IBD diagnosis , mother’s usual diet differed from the traditional diet | 70.00% | 41.67% | 54.55% |

1. Before Child's IBD diagnosis, did father's diet differ from the traditional diet

|  | CD | UC | PIBD |
| --- | --- | --- | --- |
| N= 20 |  |  |  |
| Before Child's IBD diagnosis , father’s usual diet did **not** differ from the traditional diet | 30.00% | 60.00% | 45.00% |
| Before Child's IBD diagnosis , father’s usual diet differed from the traditional diet | 70.00% | 40.00% | 55.00% |
